# Sensory Nerve Innervation of Epineurial Arterioles of the Sciatic Nerve Containing Calcitonin Gene–Related Peptide: Effect of Streptozotocin-Induced Diabetes

**DOI:** 10.1080/15438600490486732

**Published:** 2004

**Authors:** M. A. Yorek, L. J. Coppey, J. S. Gellett, E. P. Davidson

**Affiliations:** 1 Department of Internal Medicine University of Iowa and Veterans Affairs Medical Center Iowa City Iowa 52246 USA

## Abstract

The authors have determined that epineurial arterioles of the sciatic nerve are innervated by nonadrenergic, noncholinergic nerves that contribute to the regulation of vasodilation. Using immunohistochemistry, the authors determined that nerves innervating epineurial arterioles contain the neuropeptide calcitonin gene–related peptide (CGRP). Using streptozotocin-induced diabetic rats, the authors demonstrated that CGRP content in sensory nerves innervating epineurial arterioles and vasodilation in response to exogenous CGRP was decreased. In summary, epineurial arterioles of the sciatic nerve are innervated by sensory nerves containing the neuropeptide CGRP. The diabetes-like condition induced by streptozotocin reduces the content of CGRP in these nerves and exogenous CGRPmediated vasodilation. CGRP is likely an important regulator of vascular tone and compromising its function could contribute to nerve ischemia and diabetic neuropathy.


*Experimental Diab. Res.*, 5:187–193, 2004
Copyright © Taylor and Francis Inc.
ISSN: 1543-8600 print / 1543-8619 online
DOI: 10.1080/15438600490486732

### Sensory Nerve Innervation of Epineurial Arterioles of the Sciatic Nerve Containing Calcitonin Gene–Related Peptide: Effect of Streptozotocin-Induced Diabetes

**M. A. Yorek, L. J. Coppey, J. S. Gellett, and E. P. Davidson**

*Department of Internal Medicine, University of Iowa and Veterans Affairs Medical Center, Iowa City, Iowa, USA*

**The authors have determined that epineurial arterioles of the sciatic nerve are innervated by nonadrenergic, noncholinergic nerves that contribute to the regulation of vasodilation. Using immunohistochemistry, the authors determined that nerves innervating epineurial arterioles contain the neuropeptide calcitonin gene–related peptide (CGRP). Using streptozotocin-induced diabetic rats, the authors demonstrated that CGRP content in sensory nerves innervating epineurial arterioles and vasodilation in response to exogenous CGRP was decreased. In summary, epineurial arterioles of the sciatic nerve are innervated by sensory nerves containing the neuropeptide CGRP. The diabetes-like condition induced by streptozotocin reduces the content of CGRP in these nerves and exogenous CGRP-mediated vasodilation. CGRP is likely an important regulator of vascular tone and compromising its function could contribute to nerve ischemia and diabetic neuropathy.**

**Keywords** Diabetic Complications; Diabetic Neuropathy; Vasoconstriction; Vasodilation

It has been reported that noradrenergic, serotoninergic, and peptidergic nerves innervate the epineurium-perineurium vasculature of peripheral nerves, and the neurotransmitters released from these nerves regulate vascular tone and nerve blood flow [1–4]. Previously, we reported that diabetes caused impairment in acetylcholine-mediated vasodilation in epineurial arterioles of the sciatic nerve [5]. In the present study, we report that innervation by sensory nerves containing calcitonin gene–related peptide (CGRP) of epineurial arterioles of the sciatic nerve and CGRP bioactivity is altered in streptozotocin-induced diabetic rats. Impairment in innervation by sensory nerves or vascular regulation could contribute to nerve ischemia and the progression of diabetic neuropathy.

Our studies indicate that epineurial arterioles of the sciatic nerve are innervated with sensory afferent nerves containing the vasoactive peptide CGRP. CGRP is a 37–amino acid neuropeptide and a potent vasodilator [6]. Localization of CGRP receptors generally matches the CGRP peptide distribution, and in the arterial system the densities of CGRP receptors from highest to lowest has been found in the peripheral, mesenteric, femoral, and carotid arteries [6].

In diabetes, investigators have demonstrated a variety of defects associated with CGRP expression and function. In the rat ileum, it was found that after 8 weeks of diabetes, there was a decrease in CGRP immunoreactivity [7]. The same investigators found that CGRP-containing neurons were decreased in ganglion cells of the ileum and proximal colon but not decreased in the myenteric ganglia of the diabetic intestine [8]. Therefore, diabetes appears to have differential effects on CGRP expression along the intestinal tract [7–9]. In the trachea, diabetes caused a reduction in CGRP release following electrical field stimulation [10, 11]. Likewise, after 34 weeks of diabetes, there is a selective decrease in CGRP-induced relaxation in the intramural coronary arteries [12]. Lastly, Tomlinson and colleagues have

Received 29 January 2004; accepted 4 April 2004.
This work was supported by National Institute of Diabetes and Digestive and Kidney Diseases grant DK-58005 from NIH, and by a Merit Review Grant from the Veterans Affairs Administration. The contents of this manuscript are solely the responsibility of the authors and do not necessarily represent the official views of the NIH.
Address correspondence to Mark A. Yorek, 3 E 17 Veterans Affairs Medical Center, Iowa City, IA 52246, USA. E-mail: myorek@icva.gov reported that diabetes caused deficits in both anterograde and retrograde axonal transport of CGRP and a 30% to 40% decrease in the content of CGRP in the sciatic nerve, which was prevented by insulin treatment [13–15].

In the present study, we report that streptozotocin-induced diabetes of 4- to 6- and 10- to 12-week duration causes a concentration-dependent impairment of exogenous CGRP-induced vasodilation and diabetes of 10- to 12-week duration caused a decrease in CGRP levels in sensory nerves of epineurial arterioles. Because neuropeptides of sensory nerves regulate blood flow to peripheral tissues, impairment of CGRP availability and bioactivity in epineurial arterioles of the sciatic nerve may have an impact on the development of diabetes-related vascular and neural complications.

### MATERIALS AND METHODS

Unless stated otherwise, all chemicals used in these studies were obtained from Sigma Chemical Co. (St. Louis, MO).

### Animals

Male Sprague-Dawley (Harlan Sprague-Dawley, Indianapolis, IN) rats, 8 to 9 weeks of age, were housed in a certified animal care facility and food (Harlan Teklad, no. 7001, Madison, WI) and water were provided ad libitum. All institutional (approval ACURF no. 0210257) and National Institutes of Health guidelines for use of animals were followed. Diabetes was induced by intravenously injecting streptozotocin (55 mg/kg in 0.9% NaCl, adjusted to a pH 4.0 with 0.2 M sodium citrate). Control rats were injected with vehicle alone. The rats were anesthetized with halothane before injection. Diabetes was verified 48 hours later by evaluating blood glucose levels with the use of glucose-oxidase reagent strips (Lifescan, Milpitas, CA). Rats having blood glucose level of 300 mg/dL (16.7 mM) or greater were considered to be diabetic. After verification of diabetes, 2 groups of rats were established a short-term diabetic group (4 to 6 weeks' duration of diabetes) and a long-term diabetic group (10 to 12 weeks' duration of diabetes).

On the day of the experiment, nonfast blood glucose level was determined and the rats were anesthetized with Nembutal intraperitoneal (IP) (50 mg/kg, IP; Abbott Laboratories, North Chicago, IL). Afterwards, the abdominal aorta was isolated and occluded 1 to 2 cm above the branch of the common iliac artery. The rat was then sacrificed by exsanguination, and body temperature lowered with topical ice followed by isolation and removal of the epineurial arterioles for vascular studies.

### Vascular Reactivity

Videomicroscopy was used to investigate in vitro vasodilatory responsiveness of arterioles vascularizing the region of the sciatic nerve as previously described [5, 16, 17]. The epineurial arterioles used for these studies were generally oriented longitudinally in relation to the sciatic nerve; however, on occasion radially oriented vessels were also used. To isolate these vessels, the common iliac was exposed and the branch points of the internal pudendal and superior gluteal arteries identified. The vessels were then clamped, and tissue containing these vessels and its branches dissected en bloc. The block of tissue was immediately submerged in a cooled (4°C), oxygenated (20% $O_2$, 5% $CO_2$, and 75% $N_2$) Krebs Henseleit physiological saline solution (PSS) of the following composition (in mM): NaCl 118, KCl 4.7, $CaCl_2$ 2.5, $KH_2PO_4$ 1.2, $MgSO_4$ 1.2, $NaHCO_3$ 20, $Na_2$EDTA 0.026, and 5.5 glucose. Branches of the superior gluteal and internal pudendal arteries (50 to 150 $\mu$m internal diameter and 2 mm in length) were carefully dissected and trimmed of fat and connective tissue. Both ends of the isolated vessel segment were cannulated with glass micropipettes filled with PSS (4°C), and secured with 10-0 nylon Ethilon monofilament sutures (Ethicon, Cornelia, GA). The pipettes were attached to a single pressure reservoir (initially set at 0 mm Hg) under condition of no flow. The organ chamber containing the cannulated vessels was then transferred to the stage of an inverted microscope (CK2; Olympus, Lake Success, NY). Attached to the microscope were a CCTV camera (WV-BL200, Panasonic, Secaucus, NJ), video monitor (Panasonic), and a video caliper (VIA-100K; Boeckeler Instruments, Tucson, AZ). The organ chamber was connected to a rotary pump (Masterflex; Cole Parmer Instrument, Vernon Hills, IL), which continuously circulated 37°C oxygenated PSS at 30 mL/min. The pressure within the vessel was then slowly increased to 40 mm Hg. At this pressure we found that KCl gave the maximal constrictor response. Therefore, all the studies were conducted at 40 mm Hg. Internal vessel diameter (resolution of 2 $\mu$m) was measured by manually adjusting the video micrometer. After 30 minutes' equilibration, KCl was added to the bath to test vessel viability. Vessels that failed to constrict more than 30% were discarded. After washing with PSS, vessels were incubated for 30 minutes in PSS and then preconstricted with the thromboxane analog U46619 ($10^{-8}$ to $10^{-7}$ M) (Cayman Chemical, Ann Arbor, MI) to 30% to 50% of passive diameter. This vasoconstrictor agonist was selected because it is a receptor-mediated constrictor that consistently produces stable vasoconstriction in rat arterial vessels. There was no significant difference in the amount of U46619 required to induce constriction in control and diabetic vessels. Cumulative concentration-response relationships were evaluated for CGRP ($10^{-11}$ to $10^{-8}$ M), and acetylcholine ($10^{-8}$ to $10^{-4}$ M) using vessels from control and/or both groups of diabetic rats. At the end of each dose-response determination, sodium nitroprusside ($10^{-4}$ M) was added to determine maximal vasodilation.

### Immunohistochemistry

We analyzed for CGRP by immunohistochemical staining epineurial arterioles of the sciatic nerve from control and both groups of diabetic rats. Epineurial arterioles of the sciatic nerve were collected as described above with minimal preparation. Afterwards, the vessels were incubated for 30 minutes in buffer containing 1% Triton X-100 with 1% bovine serum albumin [18]. The vessels were next incubated in a mixture containing goat polyclonal antibodies against CGRP for 72 hours at 4°C. For controls, vessels were incubated in the absence of anti-CGRP. Afterwards, vessels were washed and then incubated with a mixture of Alexa-Fluor-568–conjugated donkey anti-goat immunoglobulin G (IgG) (Molecular Probes, Eugene, OR) for 24 hours at 4°C. Following the incubation with the secondary antibody, vessels were washed with 0.01 M phosphate-buffered saline (PBS), water, and then mounted with Vector-Shield. The labeled vessels derived from these studies were visualized with a Zeiss LSM 510 laser scanning confocal microscope using either 10× or 20× objectives. Optical sections along the z-axis were collected at 1.0-$\mu$m intervals at a resolution of 512 × 512. Stacks of images from slices at the same z section were combined to form a single composite image.

To confirm that CGRP levels were decreased in epineurial arterioles from diabetic rats (10- to 12-week duration), the CGRP content of intact vessels were analyzed using a CGRP (rat) radioimmunoassay (RIA) kit (Phoenix Pharmaceuticals, Belmont, CA) according to the vendor's instructions [10, 19]. To measure CGRP content of intact vessels, epineurial arterioles were pooled from 4 rats, weighed, and homogenized [10]. Levels of CGRP were reported as pg/mg wet tissue weight.

### Data Analysis

The results are presented as mean ± SEM. Dose-response curves were compared using a 2-way repeated-measure analysis of variance with autoregressive covariance structure using proc mixed program of SAS [5, 16, 17]. Whenever significant interactions were noted, specific treatment-dose-effect interactions were analyzed using a Bonferroni adjustment. A $P$ value of less .05 was considered significant.

### RESULTS

### Vascular Reactivity in Epineurial Arterioles of the Sciatic Nerve

In epineurial arterioles of the sciatic nerve from normal rats, exogenous CGRP caused vasodilation (Figure 1). CGRP-induced vascular relaxation was blocked by 1 $\mu$M $CGRP_{8-37}$, a CGRP receptor antagonist (data not shown). Data in Figure 1 also demonstrate that streptozotocin-induced diabetes caused a decrease in CGRP-mediated vasodilation (*$P < .05$ compared to control). Vasodilation induced by CGRP was impaired to a greater extent in epineurial arterioles derived from diabetic rats of 10- to 12-week duration compared to arterioles from 4- to 6-week diabetic rats ($^+P < .05$ compared to 4- to 6-week diabetic rats). In these studies the mean blood glucose level for the 4- to 6-week and 10- to 12-week diabetic rats was 412 ± 36 and 441 ± 34 mg/dL, respectively. Control rats had an average blood glucose level of 101 ± 10 mg/dL.

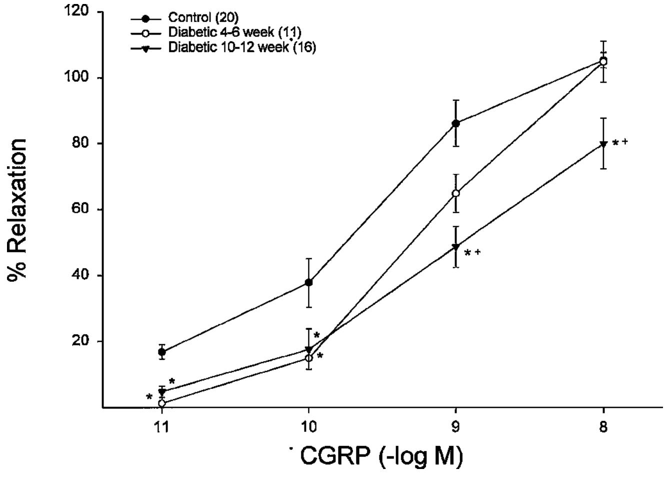

**FIGURE 1**
Determination of the effect of the duration of diabetes on CGRP-mediated vascular relaxation in epineurial arterioles of the sciatic nerve. Pressurized arterioles were preconstricted with U46619 (30% to 50%), and incremental doses of CGRP were added to the bathing solution while the steady-state vessel diameter was recorded. The data are presented as the mean ± SEM. The number of experimental observations is noted in parentheses. *The response to CGRP was significantly different compared to control rats; $^+$the response to CGRP was significantly different compared to 4- to 6-week diabetic rats.

Previously, we had demonstrated that the acetylcholine-induced vascular relaxation in epineurial arterioles of the sciatic nerve was endothelium dependent [5]. However, as demonstrated in Figure 2, CGRP-induced vascular relaxation was endothelium independent. Stripping the endothelium from epineurial arterioles of the sciatic nerve did not effect CGRP-induced vascular relaxation. In contrast, acetylcholine-mediated vascular relaxation was attenuated by removal of the endothelium.

### Immunohistochemical Staining for CGRP in Epineurial Arterioles of the Sciatic Nerve

To confirm the presence of CGRP-containing neurons in epineurial arterioles, we conducted immunohistochemical

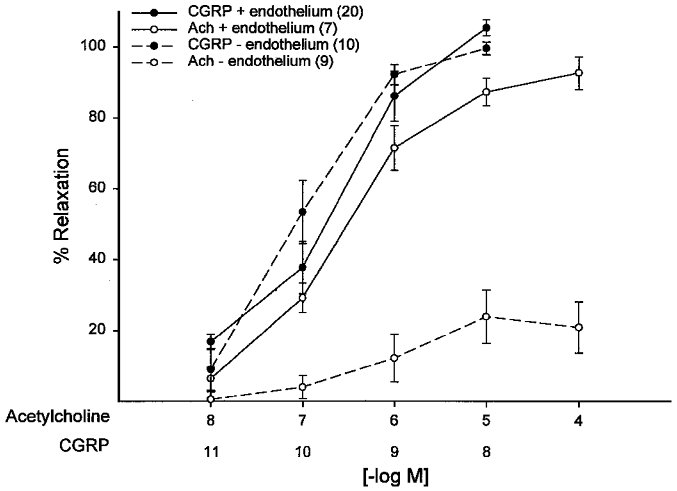

**FIGURE 2**
Determination of the effect of the endothelium on CGRP- or acetylcholine-mediated vascular relaxation in epineurial arterioles of the sciatic nerve. Epineurial arterioles were denuded, pressurized, and then preconstricted with U46619 (30% to 50%). Incremental doses of acetylcholine or CGRP were then added to the bathing solution while the steady-state vessel diameter was recorded. The data are presented as the mean ± SEM. The number of experimental observations is noted in parentheses.

staining studies using arterioles from control rats. We also examined the effect of streptozotocin-induced diabetes on the presence of CGRP-containing sensory nerves in these vessels. Data in Figure 3 demonstrate the specificity of immunostaining for CGRP in epineurial arterioles from control rats. Incubations were performed in the presence (*left panel*) or absence (*right panel*) of the primary antibody. Figure 4 present results for immunostaining for CGRP-sensory nerves in epineurial arterioles of the sciatic nerve from control (*top*) and 10- to 12-week diabetic rats (*bottom*). CGRP immunostaining was reduced in epineurial arterioles from streptozotocin-induced diabetic rats after 10 to 12 weeks of diabetes. Similar studies conducted with epineurial arterioles from 4- to 6-week diabetic rats indicated no apparent decrease in CGRP levels (data not shown). The decrease in CGRP levels in epineurial arterioles from 10- to 12-week diabetic rats was also examined using a CGRP radioimmunoassay (Figure 5). Data in Figure 5 demonstrate that the content of CGRP in sensory nerves of rat epineurial arterioles following 12 weeks of streptozotocin-induced diabetes was reduced by about 75% compared to CGRP levels in normal rats. In one experiment with epineurial arterioles from 4-week diabetic rats, the amount of CGRP present was similar to control rats (data not shown).

### DISCUSSION

These studies demonstrate that nerve fascicles innervating epineurial arterioles of the sciatic nerve are immunoreactive for CGRP. Furthermore, our studies have shown that streptozotocin-induced diabetes, 10- to 12-week duration, caused both a decrease in the CGRP content in epineurial arterioles and the ability of exogenous CGRP to induce vasodilation.

Our data demonstrate that long-term streptozotocin-induced diabetes caused a dual defect in epineurial arterioles of the sciatic nerve that would likely lead to impaired CGRP-mediated vascular function. First, we observed that diabetes caused a

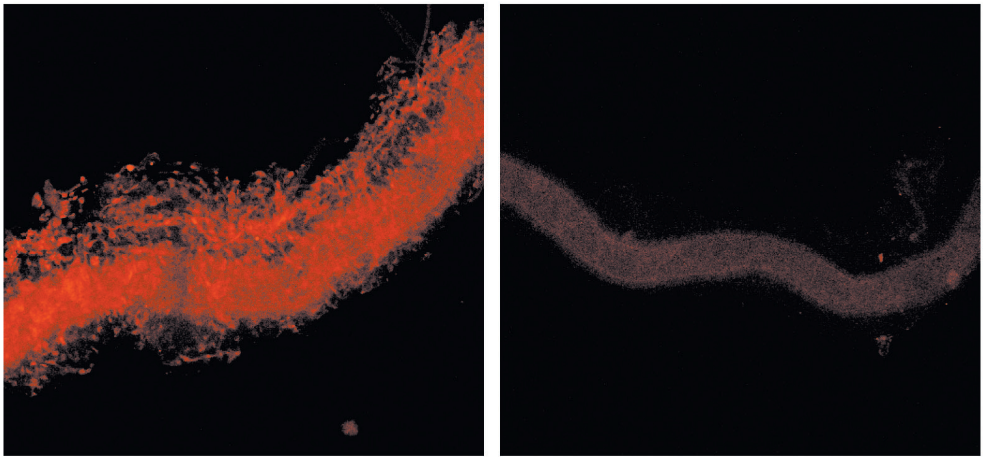

(+) anti-CGRP (-) anti-CGRP

**FIGURE 3**
Data are presented for the immunohistochemical staining of CGRP (*red*) in the presence (*left*) and absence (*right*) of the primary antibody in epineurial arterioles of the sciatic nerve from a control rat.

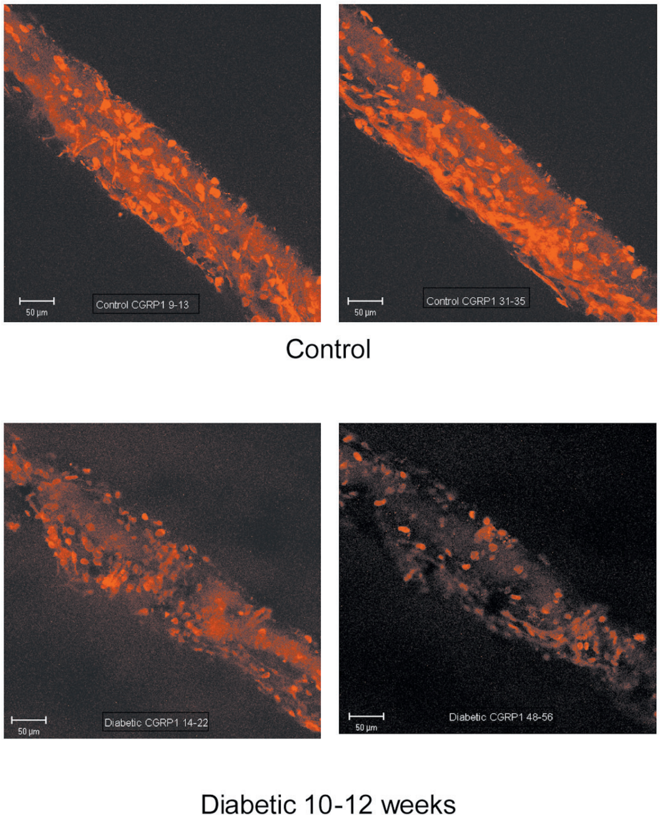

**FIGURE 4**

Determination of the effect of diabetes on CGRP content in epineurial arterioles of the sciatic nerve. Data are presented for the immunohistochemical staining of CGRP (*red*) in epineurial arterioles of the sciatic nerve from 2 separate control (*top*) and 10- to 12-week diabetic (*bottom*) rats.

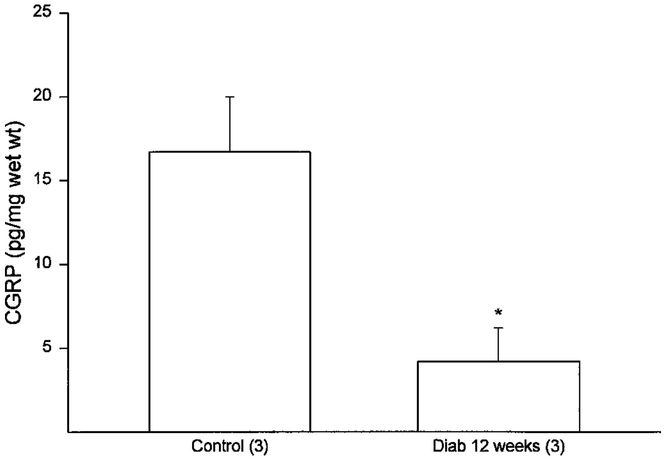

**FIGURE 5**

Determination of the effect of 12 weeks of diabetes on CGRP content in epineurial arterioles of the sciatic nerve. Epineurial arterioles from 4 control and 12-week diabetic rats were pooled and examined for CGRP content using a radioimmunoassay for rat CGRP. The data are presented as the mean ± SEM. The number of experimental observations is noted in parentheses. *The CGRP content of epineurial arterioles from 12-week diabetic rats was significantly different compared to control rats.

decrease in the level of CGRP in sensory nerves innervating epineurial arterioles of the sciatic nerve. Other investigators have also found that diabetes caused a decrease in CGRP content of the sciatic, vagus, and enteric nerves [10, 13–15, 20]. Furthermore, Rittenhouse and colleagues have reported that the synthesis of CGRP was decreased in the dorsal root ganglion of streptozotocin-induced diabetic rats [21]. Our result demonstrating that CGRP levels are decreased in long-term diabetic rats is also consistent with the studies by Zochodne and Ho [22, 23]. They demonstrated that a topical application of capsaicin caused a hyperemic response of endoneurial blood flow in control rats, which was due to a release of CGRP. However, in streptozotocin-induced diabetic rats after 4 months of hyperglycemia, the hyperemic response to capsaicin was attenuated. The decrease in CGRP levels could be due to a decrease in the transport of CGRP to nerve endings or a denervation of sensory nerves. Tomlinson and colleagues have reported that anterograde transport of CGRP in sciatic nerves was decreased in diabetes [13–15, 24]. The studies conducted by Tomlinson and colleagues focused on the entire sciatic nerve. Whether a similar defect in CGRP transport can be isolated to the sensory nerves innervating the epineurial arterioles of the sciatic nerve remains to be determined. Second, our studies have shown that exogenous CGRP-mediated vasodilation was impaired by streptozotocin-induced diabetes. Vasodilation by epineurial arterioles in response to a low dose of exogenous CGRP was impaired after 4 to 6 weeks of diabetes and at all doses of exogenous CGRP after 10 to 12 weeks of diabetes. Our results agree with the studies of Sheykhzade and colleagues that reported that long-term diabetes caused a selective depression of $\alpha$CGRP-induced relaxation in intramural coronary arteries of Wistar rats [25]. Sheykhzade and colleagues [12] suggested that the diabetes-induced impairment of CGRP-mediated vascular relaxation in coronary arteries may be due to glycosylation and/or conformational changes of the CGRP receptor. Moreover, it has been demonstrated that the vasodilatory response to CGRP involves the formation of cyclic adenosine monophosphate (cAMP) and activation of $Ca^{2+}$ signaling pathways [1, 2, 25, 26]. It is possible that the signaling mechanism(s) activated by the CGRP receptor may be impaired in diabetes, thereby causing a decrease in vasodilation in response to exogenous CGRP. This would be supported by our data, which demonstrate that at a low dose of CGRP and shorter duration

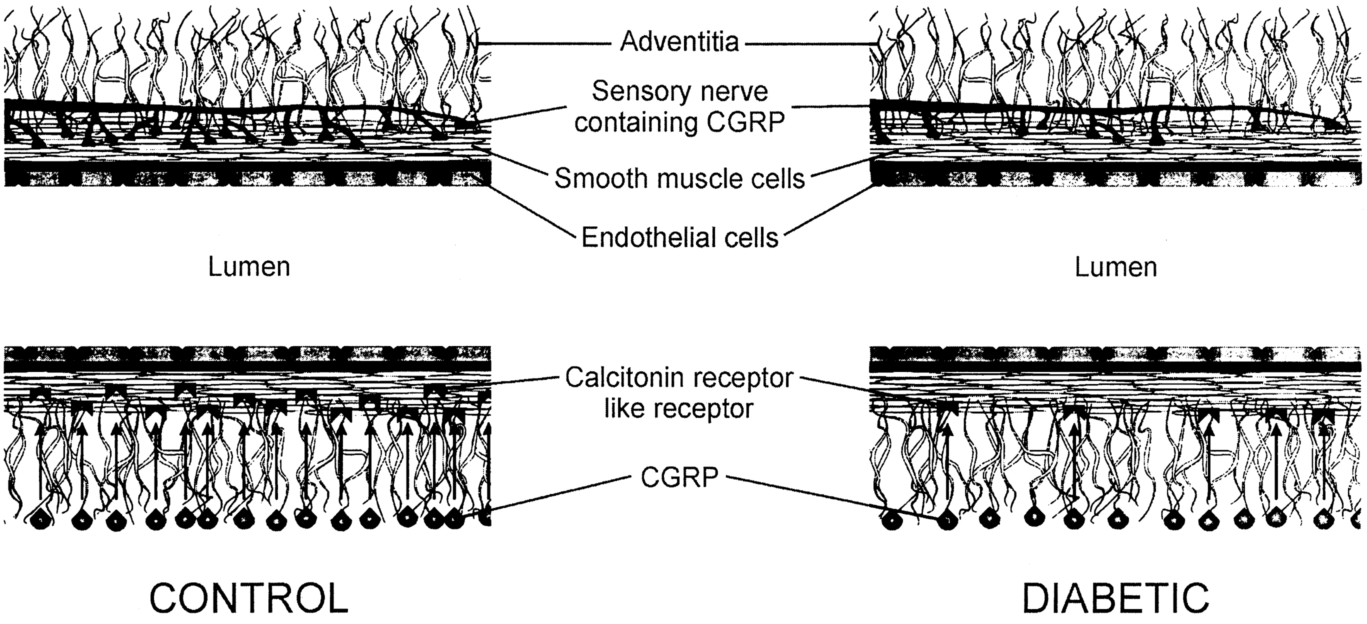

**FIGURE 6**
Representation of epineurial arterioles and innervation by sensory nerves containing CGRP: effect of diabetes.

of diabetes, vasodilation in response to exogenous CGRP is attenuated but is normal at a higher dose of CGRP. It is only after a longer duration of diabetes that vasodilation in response to all concentrations of CGRP is impaired. Additional studies will be required to address these issues.

Figure 6 provides a summary of our interpretation of the results from these studies. Epineurial arterioles of the sciatic nerve from a normal rat are innervated by sensory nerves that contain CGRP. These sensory nerves appear to be localized between the smooth muscle and adventitia. In long-term diabetic rats, the amount of CGRP present in epineurial arterioles is diminished. This could be due to a decrease in axonal transport of CGRP from the dorsal root ganglion cells or denervation of the sensory neurons. In addition to the decrease in CGRP levels in sensory nerves innervating epineurial arterioles, diabetes also causes impairment in CGRP-mediated vasodilation. Exogenous CGRP likely reacts with a calcitonin receptor–like receptor located on smooth muscle cells and causes vasodilation in epineurial arterioles by an unknown mechanism. Diabetes, in a duration sensitive manner, appears to impair this mechanism. Perhaps diabetes is causing a decrease in the number of receptors and/or impairing the signaling mechanism regulating CGRP-mediated vascular relaxation. Overall, impairment of CGRP levels and bioactivity could have an impact on the development and progression of diabetic neuropathy because the neuropeptides released by sensory nerves are thought to regulate vascular tone and blood flow to peripheral nerves [3, 4, 27, 28]. Compromising the level and function of these neuropeptides could contribute to nerve ischemia and morphological changes observed during the progression of diabetic neuropathy.

### REFERENCES

[1] Aiyar, N., Disa, J., Stadel, J. M., and Lysko, P. G. (1999) Calcitonin gene-related peptide receptor independently stimulates 3′,5′-cyclic adenosine monophosphate and $Ca^{2+}$ signaling pathways. *Mol. Cell. Biochem.,* **197,** 179–185.

[2] Herzog, M., Scherer, E. Q., Albrecht, B., Rorabaugh, B., Scofield, M. A., and Wangemann, P. (2002) CGRP receptors in the Gerbil spiral modiolar artery mediate a sustained vasodilation via a transient cAMP-mediated $Ca^{2+}$-decrease. *J. Membrane Biol.,* **232,** 1017–1025.

[3] Rechthand, E., Hervonen, A., Sato, S., and Rapoport, S.I. (1986) Distribution of adrenergic innervation of blood vessels in peripheral nerve. *Brain Res.,* **374,** 185–189.

[4] Zochodne, D. W., and Ho, L. T. (1991a). Unique microvascular characteristics of the dorsal root ganglion in the rat. *Brain Res.,* **559,** 89–93.

[5] Terata, K., Coppey, L. J., Davidson, E. P., Dunlap, J. A., Gutterman, D. D., and Yorek, M. A. (1999). Acetylcholine-induced arteriolar dilation is reduced in streptozotocin-induced diabetic rats with motor nerve dysfunction. *Br. J. Pharmacol.,* **128,** 837–843.

[6] Wimalawansa, S. J. (1996) Calcitonin gene-related peptide and its receptors: Molecular genetics, physiology, pathophysiology, and therapeutic potentials. *Endocrine Rev.,* **17,** 533–585.

[7] Belai, A., Lincoln, J., Milner, P., and Burnstock, G. (1988) Progressive changes in adrenergic, serotonergic, and peptidergic nerves in proximal colon of streptozotocin-diabetic rats. *Gastroenterology,* **95,** 1234–1241.

[8] Belai, A., and Burnstock, G. (1990) Changes in adrenergic and peptidergic nerves in the submucous plexus of streptozotocin-diabetic rat ileum. *Gastroenterology,* **98,** 1427–1436.

[9] Belai, A., and Burnstock, G. (1987) Selective damage of intrinsic calcitonin gene-related peptide-like immunoreactive enteric nerve fibers in streptozotocin-induced diabetic rats. *Gastroenterology,* **92,** 730–734.

[10] Calcutt, N. A., Chen, P., and Hua, X. Y. (1998) Effects of diabetes on tissue content and evoked release of calcitonin gene-related peptide-like immunoreactivity from rat sensory nerves. *Neurosci. Letts.,* **254,** 129–132.

[11] Nemeth, J., Szilvassy, Z., Than, M., Oroszi, G., Sari, R., and Szolcsanyi, J. (1999) Decreased sensory neuropeptide release from trachea of rats with streptozotocin-induced diabetes. *Eur. J. Pharmacol.,* **369,** 221–224.

[12] Sheykhzade, M., Dalsgaard, G. T., Johansen, T., and Nyborg, N. C. B. (2000) The effect of long-term streptozotocin-induced diabetes on contractile and relaxation responses of coronary arteries: Selective attenuation of CGRP-induced relaxations. *Br. J. Pharmacol.,* **129,** 1212–1218.

[13] Diemel, L. T., Steves, E. J., Willars, G. B., and Tomlinson, D. R. (1992) Depletion of substance P and calcitonin gene-related peptide in sciatic nerve of rats with experimental diabetes: effects of insulin and aldose reductase inhibition. *Neurosci. Letts.,* **137,** 253–256.

[14] Tomlinson, D. R., Fernyhough, P., and Diemel, L. T. (1996) Neurotrophins and peripheral neuropathy. *Phil. Trans. R. Soc. Lond.,* **351,** 455–462.

[15] Tomlinson, D. R., Fernyhough, P., and Diemel, L. T. (1997) Role of neurotrophins in diabetic neuropathy and treatment with nerve growth factors. *Diabetes,* **46,** S43–S49.

[16] Coppey, L. J., Davidson, E. P., Dunlap, J. A., Lund, D. D., and Yorek, M. A. (2000) Slowing of motor nerve conduction velocity in streptozotocin-induced diabetic rats is preceded by impaired vasodilation in arterioles that overlie the sciatic nerve. *Int. J. Exp. Diabetes Res.,* **1,** 131–143.

[17] Coppey, L. J., Gellett, J. S., Davidson, E. P., Dunlap, J. A., Lund, D. D., and Yorek, M. A. (2001) Effect of antioxidant treatment of streptozotocin-induced diabetic rats on endoneurial blood flow, motor nerve conduction velocity and vascular reactivity of epineurial arterioles of the sciatic nerve. *Diabetes,* **50,** 1927–1937.

[18] Qiu, J., Steyger, P. S., Trune, D. R., and Nuttall, A. L. (2001) Co-existence of tyrosine hydroxylase and calcitonin gene-related peptide in cochlear spiral modiolar artery of guinea pigs. *Hearing Res.,* **155,** 152–160.

[19] Zochodne, D. W., Allison, J. A., Ho, W., Ho, L. T., Hargreaves, K., and Sharkey, K. A. (1995) Evidence for CGRP accumulation and activity in experimental neuromas. *Am. J. Physiol.,* **268,** H584–H590.

[20] Belai, A., Lincoln, J., and Burnstock, G. (1987) Lack of release of vasoactive intestinal polypeptide and calcitonin gene-related peptide during electrical stimulation of enteric nerves in streptozotocin-diabetic rats. *Gastroenterology,* **93,** 1034–1040.

[21] Rittenhouse, P. A., Marchand, J. E., Chen, J., Kream, R. M., and Leeman, S. E. (1996) Streptozotocin-induced diabetes is associated with altered expression of peptide-encoding mRNAs in rat sensory neurons. *Peptides,* **17,** 1017–1022.

[22] Zochodne, D. W., and Ho, L. T. (1991b) Influence of perivascular peptides on endoneurial blood flow and microvascular resistance in the sciatic nerve of the rat. *J. Physiol.,* **444,** 615–630.

[23] Zochodne, D. W., and Ho, L. T. (1993) Diabetes mellitus prevents capsaicin from inducing hyperaemia in the rat sciatic nerve. *Diabetologia,* **36,** 493–496.

[24] Riaz, S. S., and Tomlinson, D. R. (1999) Clenbuterol stimulates neurotrophic support in streptozotocin-diabetic rats. *Diabetes Obes. Metab.,* **1,** 43–51.

[25] Sheykhzade, M., Berg, M., and Nyborg, N. C. (2001) Mechanism of CGRP-induced relaxation in rat intramural coronary arteries. *Br. J. Pharmacol.,* **132,** 1235–1246.

[26] Champion, H. C., Bivalacqua, T. J., Pierce, R. L., Murphy, W. A., Coy, D. H., Hyman, A. L., and Kadowitz, P. J. (2003) Responses to human CGRP, ADM, and PAMP in human thymic arteries. *Am. J. Physiol. Integr. Comp. Physiol.,* **284,** R531–R537.

[27] Appenzeller, O., Dhital, K. K., Cowen, T., and Burnstock, G. (1984) The nerves to blood vessels supplying blood to nerves: The innervation of vasa nervorum. *Brain Res.,* **304,** 383–386.

[28] Kihara, M., and Low, P. A. (1990). Regulation of rat nerve blood flow: Role of epineurial alpha-receptors. *J. Physiol.,* **422,** 145–152.

